# Feature Selection with Neighborhood Entropy-Based Cooperative Game Theory

**DOI:** 10.1155/2014/479289

**Published:** 2014-08-25

**Authors:** Kai Zeng, Kun She, Xinzheng Niu

**Affiliations:** School of Computer Science and Engineering, University of Electronic Science and Technology of China, Chengdu 611731, China

## Abstract

Feature selection plays an important role in machine learning and data mining. In recent years, various feature measurements have been proposed to select significant features from high-dimensional datasets. However, most traditional feature selection methods will ignore some features which have strong classification ability as a group but are weak as individuals. To deal with this problem, we redefine the redundancy, interdependence, and independence of features by using neighborhood entropy. Then the neighborhood entropy-based feature contribution is proposed under the framework of cooperative game. The evaluative criteria of features can be formalized as the product of contribution and other classical feature measures. Finally, the proposed method is tested on several UCI datasets. The results show that neighborhood entropy-based cooperative game theory model (NECGT) yield better performance than classical ones.

## 1. Introduction

With the development of information acquirement, more and more high-dimensional data need to be processed for some real world applications [1, 2]. Nevertheless, some of the features in huge datasets are irrelevant or redundant, which lead classification algorithms to low efficiency and overfitting. How to identify the most characterizing features [3–5] is critical to reduce the classification error and increase classifier's computation speed. Thus, feature selection as a common technique used in data preprocessing for the classification algorithms has attracted much attention in recent years [2].

Up to present, some different information theoretical-based selectors are employed in feature selection, such as mutual information (MI) [6], rough set (RS) [1, 7], and mRMR [2]. The main idea of those methods is to find the significant features for classification by calculating the significance of individual feature. In [8, 9], the authors pointed out that the classical methods ignore features which as a group have strong discriminatory power but are weak as individuals. As a result, those traditional methods are unable to deal with some practical problems, such as feature cooccurrences [10].

Aimed at this problem in feature selection, Guyon and Elisseeff [11] constructed an example to illuminate that two variables which are useless by themselves can be useful together. Consequently, how to evaluate the correlation of features is another important aspect besides estimating the classification ability of the individual feature. Cohen et al. proposed the concept of feature contribution in the feature subset to describe the correlation of features via cooperative game theory [12]. One drawback of the method is their less generalization of the selected features on other classifiers, because they are tightly coupled with specified learning algorithms. Sun et al. [8, 9] used Shannon's entropy to define the independent, redundant, and interdependent features. Then the interdependent features are used for calculating the feature contribution under the framework of cooperative game theory. A universal feature selection method was proposed in [8, 9] which can be used in conjunction with many traditional feature selection methods. It is a pity that Shannon's entropy is only suitable for dealing with nominal data, such as male or female and good or bad. If the attributes are numerical or set-valued, researchers generally adopt discretization technique to transform the nonnominal to the nominal, which would bring loss of information inevitably [13]. It is obviously unreasonable to measure similarity or dissimilarity with Euclidean distance as to categorical attributes in numerical methods.

Thus, in summary, the method of feature selection still needs improvement because of the following problems.

1. Most of traditional methods, such as MI, RS, and mRMR, ignore the interdependent features which seemed without direct effects on decision.
2. Although CoFS [9] can be used for mining the interdependent features, this kind of Shannon's entropy-based method would bring loss of information inevitably during the process of discretization. It can lead to computational deviation because of the distortion of the datasets.

Hu et al. investigated neighborhood entropy to avoid data discretization [6]. It makes a breakthrough in this problem. Based on the theory of cooperative game and the concept of neighborhood entropy, the contribution of this paper includes the following: (1) we redefine the redundancy, interdependence, and independence of features by using neighborhood entropy to avoid the discretization; (2) moreover, the neighborhood entropy-based feature contribution is presented to handle the feature selection problem under the framework of cooperative game; and (3) the proposed method is tested on the UCI datasets. The results show that neighborhood entropy-based cooperative game theory model (NECGT) yield better performance than classical ones.

The paper is organized as follows: in Section 2, some basic concepts about feature selection, neighborhood, and game theory are briefly reviewed. In Section 3, the NECGT model is investigated in detail.  Section 4 shows the application of NECGT for feature evaluating and feature selection. Numeric experiments are reported in Section 5. Finally, Section 6 concludes the paper.

## 2. Preliminaries

In this section, the formalism of feature selection is presented. The common concepts about neighborhood entropy and cooperative game theory are introduced.

### 2.1. Feature Selection

**Definition 1 .**

Knowledge representation is realized via the information system (IS) which is a tabular form, similar to databases. An information system is IS = (*U*, *C*, *D*), where *U* = {*x*_1_, *x*_2_,…, *x*_*n*_} is a nonempty finite set of objects, *C* is a nonempty finite set of conditional attributes, and *D* is the decision attribute which represents the target classes. The goal of feature selection is to find the minimum subset *S* from set *C*. The subset *S* is optimized for the performance of machine learning algorithm.

### 2.2. Neighborhood Entropy-Based Measurements

Evaluating relevance between features (attributes, variables) is an important task in pattern recognition and machine learning. Shannon's entropy and mutual information provide intuitive tools to measure the uncertainty of random variables and the information shared by two different features in discrete spaces. However, there is a limitation in computing relevance between numerical features with mutual information due to problems of loss of information in the process of discretization. In [6], the authors integrate the concept of neighborhood into Shannon's information theory and propose a new information measure, called neighborhood entropy.

**Definition 2 .**

For all *x*_*i*_ ∈ *U*, *δ* ≥ 0, we say *δ*(*x*_*i*_) is a *δ* neighborhood of *x*_*i*_ whose centre is *x*_*i*_ and radius is *δ*, where

(1) $$\begin{array}{c} \delta \left(x_{i}\right)=\left\{y\;|\;\Delta \left(x_{i},y\right)\leq \delta ,y\in U\right\}. \end{array}$$

**Definition 3 .**

Let IS = (*U*, *C*, *D*) be an information system, and Δ is a given distance function. We say (*U*, *δ*) is a neighborhood approximation space when the following conditions are met

1. Δ(*x*_*i*_, *x*_*j*_) ≥ 0 if and only if *x*_*i*_ = *x*_*j*_, Δ(*x*_*i*_, *x*_*j*_) = 0;
2. Δ(*x*_*i*_, *x*_*j*_) = Δ(*x*_*j*_, *x*_*i*_);
3. Δ(*x*_*i*_, *x*_*k*_) ≤ Δ(*x*_*i*_, *x*_*j*_) + Δ(*x*_*j*_, *x*_*k*_).

**Remark 4 .**

To deal with nominal attributes and numerical attributes, which are common in practice, we use an extended Euclidean distance as the method introduced in literature [13]. The distance function Δ is computed as follows.

Let IS = (*U*, *C*, *D*) be an information system, *a*_*l*_ ∈ *C*

(2) Δ(xi,xj)=∑l=1Ndal(xi,xj)2,dal(xi,xj)  ={nom⁡al(xi,xj)if  al  is  a  nominal  attributenumal(xi,xj)if  al  is  a  numerical  attribute,

where

(3) $$\begin{array}{c} {\mathrm{nom}}_{a_{l}}\left(x_{i},x_{j}\right)=\{\begin{array}{cc} 0 & \text{if}\;\;x_{i}^{l}=x_{j}^{l}\\ 1 & \text{if}\;\;x_{i}^{l}\neq x_{j}^{l} \end{array} \end{array}$$

and num_*a*_*l*__(*x*_*i*_, *x*_*j*_) = |*x*_*i*_^*l*^ − *x*_*j*_^*l*^|.

Entropy is a key measure for information. Since it is capable of quantifying the uncertainty of random variables and scaling the amount of information shared by them effectively, it has been widely used in many fields [6, 14].

**Definition 5 .**

Let IS = (*U*, *C*, *D*) be an information system, where *U* = {*x*_1_, *x*_2_,…, *x*_*n*_} is described by the features *C* and *D*. *S*⊆*C* is a subset of attributes. The neighborhood of sample *x*_*i*_ is denoted by *δ*_*s*_(*x*_*i*_). Then the neighborhood uncertainty of the sample *x*_*i*_ is defined as

(4) $$\begin{array}{c} {\text{NE}}_{\delta }^{x_{i}}\left(S\right)=-\log_{2}\frac{\left|\delta_{S}\left(x_{i}\right)\right|}{n}, \end{array}$$

where |*δ*_*S*_(*x*_*i*_)| is cardinality of set *δ*_*S*_(*x*_*i*_).

The average uncertainty of the set of samples is computed as

(5) $$\begin{array}{c} {\text{NE}}_{\delta }\left(S\right)=-\frac{1}{n}\overset{n}{\underset{i=1}{\sum }}\log_{2}\frac{\left|\delta_{S}\left(x_{i}\right)\right|}{n}. \end{array}$$

**Definition 6 .**

Let IS = (*U*, *C*, *D*) be an information system. *R* and *S* are two subsets of attributes. The neighborhood of sample *x*_*i*_ in feature subspace *S* ∪ *R* is denoted by *δ*_*S*∪*R*_(*x*_*i*_); then the joint neighborhood entropy NE(*R*, *S*) is computed as

(6) $$\begin{array}{c} {\text{NE}}_{\delta }\left(R,S\right)=-\frac{1}{n}\overset{n}{\underset{i=1}{\sum }}\log_{2}\frac{\left|\delta_{R\cup S}\left(x_{i}\right)\right|}{n}. \end{array}$$

**Definition 7 .**

Let IS = (*U*, *C*, *D*) be an information system. *R* and *S* are two subsets of attributes. The conditional neighborhood entropy of *R* to *S* is defined as

(7) $$\begin{array}{c} {\text{NE}}_{\delta }\left(R\;|\;S\right)={\text{NE}}_{\delta }\left(R,S\right)-{\text{NE}}_{\delta }\left(S\right). \end{array}$$

Conditional entropy refers to the uncertainty of *R* when *S* is known. From this definition, if *R* completely depends on *S*, then NE_*δ*_(*R*∣*S*) is zero. This means that no more information is required to describe *R* when *S* is known. Otherwise, NE_*δ*_(*R*∣*S*) = NE_*δ*_(*R*) denotes that knowing *S* will do nothing to observe *R*.

To quantify how much information is shared by two features *R* and *S*, a concept termed neighborhood mutual information NMI(*R*; *S*) is described as follows.

**Definition 8 .**

Let IS = (*U*, *C*, *D*) be an information system. *R* and *S* are two different subsets of attributes. NMI(*R*; *S*) is defined as

(8) $$\begin{array}{c} {\text{NMI}}_{\delta }\left(R;S\right)={\text{NE}}_{\delta }\left(R\right)+{\text{NE}}_{\delta }\left(S\right)-{\text{NE}}_{\delta }\left(R,S\right). \end{array}$$

**Remark 9 .**

From this definition, the neighborhood mutual information becomes a measurement of relevance of features. The value of NMI(*R*; *S*) will be very high, if *R* and *S* are closely related with each other; otherwise, NMI(*R*; *S*) = 0 denotes that these two features are totally unrelated. As it is well-known, mutual information is widely applied in evaluating the significance of features when *S*⊆*C* and *R* = *D*. It reflects how much information is shared by subsets of conditional features *S* and decision feature *D*.

**Definition 10 .**

Let IS = (*U*, *C*, *D*) be an information system. *R*, *S*, and *W* are three subsets of attributes. The conditional mutual information of *R* and *S* is defined as

(9) $$\begin{array}{c} {\text{NMI}}_{\delta }\left(R;S\;|\;W\right)={\text{NE}}_{\delta }\left(R\;|\;W\right)-{\text{NE}}_{\delta }\left(R\;|\;S,W\right). \end{array}$$

The conditional mutual information represents the quantity of information shared by *R* and *S* when *W* is known. That is to say, NMI_*δ*_(*R*; *S*∣*W*) implies that *S* brings information about *R* which is not already contained in *W*.

### 2.3. Cooperative Game Theory

Cooperative game theory introduces the concept of coalitional games in which a set of players are associated with a real function that denotes the payoff achieved by different subcoalitions in a game.

**Definition 11 .**

A cooperative game is defined by a pair (*N*, *μ*), where *N* = {1,…, *n*} is the set of all players and *μ*(*S*), for every *S*⊆*N*, is a real number associating a worth with the coalition *S*.

Game theory further pursues the question of representing the contribution of each player to the game by constructing a worth function *μ*(*S*), which assigns a real value to each player. The values correspond to the contribution of the players in achieving a high payoff. Banzhaf value [15] was proposed by Banzhaf, which yields a unique outcome in cooperative games, to measure the contribution of players in the game [8, 9]. It is based on counting, for each player, the number of coalitions to which the player is crucial to winning [9]. In our study, we use this measurement to evaluate the contribution of features.

## 3. Neighborhood Entropy-Based Cooperative Game Theory Model

Conventional feature selection algorithms tend to select features which has high relevance with the target class and low redundancy among the selected features. The major disadvantage of these algorithms is that they ignored the dependencies between the candidate feature and unselected features. For example, mRMR [2] introduced the criterion, namely, “Min-Redundancy,” to eliminate the redundant features. However, the authors in [8, 9] pointed out that it is likely to disregard the intrinsic interdependent groups which as a group have strong discriminatory power but are weak as individuals. The main reason is that features which have been labeled “redundancy” are in reality interdependent to the selected feature subset [9].

In this work, neighborhood entropy-based measurements are adapted to distinguish the relationship of redundancy, interdependence, and independence between features. Then, we use Banzhaf value to computing the contributions of each feature. A universal framework to evaluate the significance of features is investigated.

### 3.1. Neighborhood Entropy-Based Redundancy, Interdependence, and Independence Analysis for Features

#### 3.1.1. Redundancy

**Definition 12 .**

A conditional feature *C*_*j*_ is said to be redundant with *C*_*i*_ if the relevance between *C*_*j*_ and target class *D* will be reduced under the condition of *C*_*i*_. The formulation is defined as follows:

(10) $$\begin{array}{c} {\text{NMI}}_{\delta }\left(C_{j};D\;|\;C_{i}\right)<{\text{NMI}}_{\delta }\left(C_{j};D\right). \end{array}$$

Redundancy means that there is redundant information shared between *C*_*j*_ and target class *D* when *C*_*i*_ is known.

#### 3.1.2. Interdependence

**Definition 13 .**

Suppose *C*_*i*_ and *C*_*j*_ are interdependent on each other, then the relevance between *C*_*j*_ and target class *D* will be increased conditioned by *C*_*i*_. Thus, two features *C*_*i*_ and *C*_*j*_ are interdependent on each other if the following form is satisfied:

(11) $$\begin{array}{c} {\text{NMI}}_{\delta }\left(C_{j};D\;|\;C_{i}\right)>{\text{NMI}}_{\delta }\left(C_{j};D\right). \end{array}$$

According to the explanation about redundancy, interdependence means that the amount of information shared between *C*_*j*_ and target class *D* will be increased when *C*_*i*_ is known. In another word, the impact of each feature on the classification performance cannot be ignored and replaced.

#### 3.1.3. Independence

**Definition 14 .**

If two features *C*_*i*_ and *C*_*j*_ are completely independent, then the relevance between target class *D* and any one of them will not be changed by the other emerging as a condition. That is,

(12) $$\begin{array}{c} {\text{NMI}}_{\delta }\left(C_{j};D\;|\;C_{i}\right)={\text{NMI}}_{\delta }\left(C_{j};D\right). \end{array}$$

Based on the definitions above, it can be concluded that some interdependent features, which seem to unimportant to the decision, should be considered in the selection process. We will discuss this problem in detail in the next section.

### 3.2. Feature Evaluation Framework Based on Cooperative Game Theory

In [15], Banzhaf proposed that a winning coalition is one for which *μ*(*S*) = 1 and a losing coalition is one for which *μ*(*S*) = 0. Each coalition *S* ∪ {*i*} that wins when *S* loses is called a swing for player *i* [8, 9]. That is, Δ_*i*_(*S*) = *μ*(*S* ∪ *i*) − *μ*(*S*) = 1. It means that the membership of player *i* in the coalition is crucial to the coalition winning. In other words, the greater the number of swings for player *i*, the more important the player *i*.

Then Banzhaf value is defined as

(13) $$\begin{array}{c} \phi_{B}\left(i\right)=\frac{1}{2^{n}-1}\underset{S\subseteq N\setminus i}{\sum }\Delta_{i}\left(S\right), \end{array}$$

where Δ_*i*_(*S*) = *μ*(*S* ∪ *i*) − *μ*(*S*).

The Banzhaf value can be interpreted as the average contribution of player *i* alone to all coalitions [16].

The Banzhaf value measures the distribution of power among the players in the voting game, which can be transformed into the arena of feature selection [8, 9]. In the feature selection game, every feature can be regarded as a player. Thus, the Banzhaf value can be used to estimate the contribution of each feature.

From the definition of interdependence, it is easy to see that the optimal feature subset is the one in which all the features are relevant to the target class and interdependent on each other [8]. Given a candidate subset coalition *S*, the feature *C*_*i*_  (*C*_*i*_ ∉ *S*) is to be estimated. Let ID_*S*_^*i*^ be the number of features which fall into interdependent relationship with the feature *C*_*i*_. The contribution of the feature *C*_*i*_ on coalition *S* can be redefined as the following description:

(14) $$\begin{array}{c} \Delta_{i}\left(S\right)=\{\begin{array}{cc} 1 & \;\text{NM}{\text{I}}_{\delta }\left(S;D\;|\;C_{i}\right)\geq 0,{\text{ID}}_{S}^{i}\geq \frac{\left|S\right|}{2}\\ 0 & \text{else} \end{array} \end{array}$$

which means that the feature is crucial to win the coalition only if it both increases the relevance of the unitary subset *S* on the target class and is interdependent with at least half of the members. Furthermore, we can get the average contribution of player *i* to all coalitions according to the Banzhaf value. The definition about Δ_*i*_(*S*) is similar as the formula (11) in [8]. Nevertheless, we use neighborhood conditional mutual information NMI_*δ*_(*S*; *D*∣*C*_*i*_) rather than Shannon's conditional mutual information in [8]. The neighborhood entropy-based method can avoid discretization of the samples.

The traditional feature selection methods were proposed based on some feature measures [2, 6, 13], such as mutual information (MI), rough set (RS), and mRMR. These measures were usually used in evaluating the significance of features. Actually, this type of significance can only be called the significance for decision (SIGFD). In the framework of neighborhood entropy-based cooperative game theory (NECGT), the contribution of one feature in the coalitions is another important aspect. Here, we give the neighborhood entropy-based formulaic feature measure according to [9]:

(15) $$\begin{array}{c} \text{SIG}\left(i\right)=\phi_{B}\left(i\right)\times \text{SIGFD}\left(i\right), \end{array}$$

where SIGFD(*i*) can be any of traditional feature measures and *ϕ*_*B*_(*i*) is the Banzhaf value.

## 4. Feature Selection Algorithm with NECGT

Before giving the algorithm of feature selection, details of the feature contribution evaluation method based on the Banzhaf value are presented in Algorithm 1.

An information system is IS = (*U*, *C*, *D*), where *U* is a nonempty finite set of objects, *C* is a nonempty finite set of conditional attributes, and *D* is the decision attribute which represents the target classes. The output of this evaluation framework is a vector *Ω* of which each element *Ω*(*i*) represents the Banzhaf value *ϕ*_*B*_(*i*) of feature *C*_*i*_.

In fact, it is impractical to get the optimal subset of features from 2^*n*^ − 1 candidates through exhaustive search, where *n* is the number of features. The greedy search guided by some heuristics is usually more efficient than the plain brute-force exhaustive search. A forward search algorithm for feature selection with NECGT is written as shown in Algorithm 2.

In the forward greedy search, one starts with an empty set of attributes and keeps adding features to the subset of selected attributes one by one. Each selected attribute maximizes the significance of the current subset. This selection procedure will be terminated if the number of selected features is larger than the user-specified threshold *ε*. Without loss of generality, to handle the feature selection problem, a general significance to decision SIGFD(*i*) is presented by employing any classical criteria, such as MI and mRMR.

It is worth noting that the calculation of the Banzhaf value requires summing over all possible subsets of features, which can extremely increase the computational complexity of Algorithm 1. In fact, it is impossible to consider all coalitions for features, especially large coalitions. In [9], the author proposed that the number of features correlated with a certain feature is much smaller than the total number of features in the real datasets. Thus, we use a limit value *λ* being a bound on the coalition size. The Banzhaf value can be redefined as

(16) $$\begin{array}{c} \phi_{B}^{\lambda }\left(i\right)=\frac{1}{\Pi_{\lambda }}\underset{S\subseteq \Pi_{\lambda }}{\sum }\Delta_{i}\left(S\right), \end{array}$$

where Π_*λ*_ is the set of subsets of feature set *N*∖*i* limited by *λ*. The usage of bounded sets coupled with the method for the Banzhaf value estimation yields an efficient and robust way to estimate the contribution of a feature to the task of feature selection. In our study, *λ* is set to $\sqrt{N}$ according to [17], where *N* is the number of features.

## 5. Experiments

In this section, we will evaluate the proposed model NECGT by a series of experiments. In this study, method of feature selection has improved from two aspects as follows.

1. The concept of neighborhood entropy-based feature contribution is proposed to avoid the process of discretization which would bring loss of information.
2. The neighborhood entropy-based feature contribution is used for feature selection. It can enhance the feature selection ability.

Hence, we design two experiments to verify the two points above. To compare the effectiveness of NECGT, we employ two popular feature selectors: RS [13] and mRMR [2], for evaluating the significance to decision (SIGFD). This experiment can be called NECGT, SIGFD versus SIGFD. On the other hand, we choose CoFS [9] as the benchmark. This Shannon's entropy-based method can also be used to compute feature contribution. However, it must adopt discretization technique to preprocess data. This experiment is called NECGT versus CoFS where the SIGFD is MI [6].

The datasets are downloaded from the UCI Machine Learning Repository (http://www.ics.uci.edu/~mlearn/). They are described in Table 1.

The numerical attributes of the samples are linearly normalized within [0,1]. Three popular leaning algorithms such as CART, liner SVM, and RBF-SVM are introduced to evaluate the quality of selected features. The experiments were run in a 10-fold cross validation mode. The parameters of the linear SVM and RBF-SVM are taken as the default values (the use of the MATLAB toolkit osu_svm3.00). Literature [1] has explained that the result is optimal if threshold *δ* is set between 0.1 and 0.2. In the experiment, threshold *δ* is set to 0.15 in our method.

### 5.1. Experiment 1: NECGT-SIGFD versus SIGFD

First of all, we give an example to show the difference between NECGT-SIGFD and SIGFD in detail. mRMR is employed as the metric of significance to decision (SIGFD). All samples in Glass are used in this test where learning algorithm RBF-SVM (RSVM) is chosen to evaluate the selected feature subsets.

**Figure 1:**
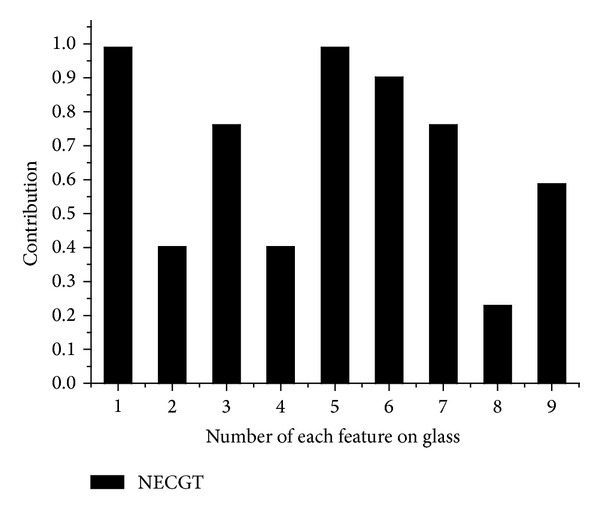
The contribution of each feature on glass.

**Figure 2:**
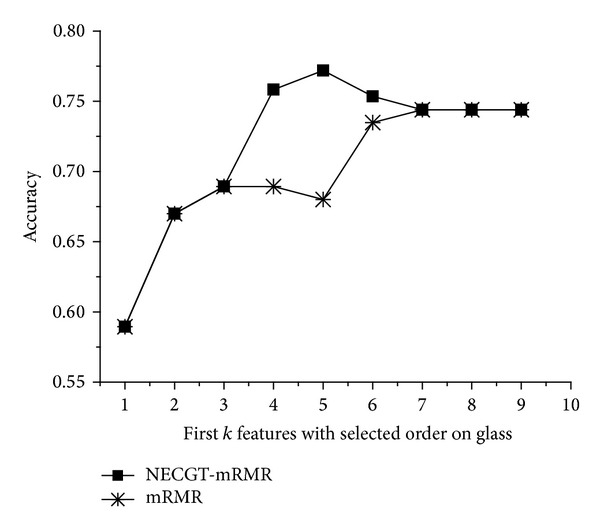
The results of NECGT-mRMR versus mRMR.

The order of the features, which are kept being added to the feature space, is shown in Table 2. There are altogether 9 features in glass. The main differences are the feature subsets from 3rd to 7th where the order selected by mRMR is 2, 3, 8, 1, 6 and the order selected by NECGT-mRMR is 3, 1, 6, 2, 8. mRMR regards the features 1 and 6 as unimportant individuals to the decision. However, the two features get high contribution scores which are showed in Figure 1. Meanwhile, we see that the contributions of features 2 and 8 are relatively less. It can be inferred that features 1 and 6 are more competitive than features 2 and 8 in a feature group. Then, we compare the classification accuracy of the feature subset to verify the inference. In Figure 2, the number *k* on *x*-axis of figures refers to the first *k* features with selected order (as is shown in Table 2) by different selectors. The *y*-axis represents the performance of classifiers of the first *k* features. The classification accuracy of raw data is 74.4%. No matter what mRMR groups the features, it still cannot surpass 74.4%. Whereas in the view of NECGT-mRMR, the feature subsets 4, 7, 3, 1, and 6 can reach up to 77.19% because of the high contribution of features 1 and 6. The theory of cooperative game emphasizes the coactions of the features. Obviously, the features 1 and 6 greatly enhance the discriminatory power of the attribute groups 4, 7, and 3 although they are weak as individuals. In contrary, the competent individual features, such as 2 and 8, are not necessarily well performed in the view of cooperative game theory.

For further comparison, the effectiveness of NECGT-SIGFD is measured by the classification performance on different datasets besides glass. We build classification models with the selected features and test their classification performance. mRMR and RS are chosen as SIGFD.

Sun et al. [9] proposed the concept about “acceptable” numbers of selected features to verify the effectiveness of the features selection algorithm. The “acceptable” number means that about a third of original features remained for a dataset. This method is also used in our study. The selected features with different algorithms are presented in Table 3. The orders of the features in the tables are the orders that the features are kept being added to the feature space. We compare the raw data, NECGT-mRMR, mRMR, NECGT-RS, and RS in Tables 4, 5, and 6, where learning algorithms are CART, linear SVM, and RBF SVM, respectively. The last column of Tables 4–6 records the average efficiency value of these different feature selection models. The feature subsets selected by NECGT-SIGFD and SIGFD are different. Just as the explanation on glass datasets, NECGT-SIGFD prefers to the feature which is likely to bring better overall performance for the feature coalition instead of individual effect. The comparison of win/tie/loss between NECGT-SIGFD and SIGFD is 20/5/5.  Table 7 summarizes NECGT-SIGFD model which yields better performance than SIGFD model in most cases. It means that the features subsets selected by NECGT-SIGFD have strong discriminatory power as a group. Meanwhile, we also find that SIGFD yields better performance than NECGT-SIGFD in a small number of cases. As is known to all, the data sample is unpredictable in a real-world environment. As a result, the interdependent features may not even exist in some datasets. It is difficult to guarantee that our method is always efficient. Nonetheless, the method suggested an effective way to retain useful interdependent features and groups as many as possible.

### 5.2. Experiment 2: NECGT versus CoFS

Both of NECGT and CoFS [9] can be used to estimate the contribution of features. In our study, the feature contribution measurement is defined based on neighborhood entropy. The CoFS method is based on Shannon's entropy. This is the contribution of our research compared to CoFS in [9]. It is important to note that we calculate the feature contribution under the framework of cooperative game theory same as [9]. The datasets are discretized for CoFS, whereas NECGT deals with original samples directly. Discretization can lead to computational deviation of contribution evaluation. Conversely, neighborhood entropy-based method NECGT will make a fairly good treatment on the issue of information losing by avoid discretization. Then the experiment on Lymphography and Wpbc is given to prove the validity of the proposed method by comparing NECGT with CoFS. MI [6] is employed as the metric of significance to decision (SIGFD) in this experiment.

**Figure 3:**
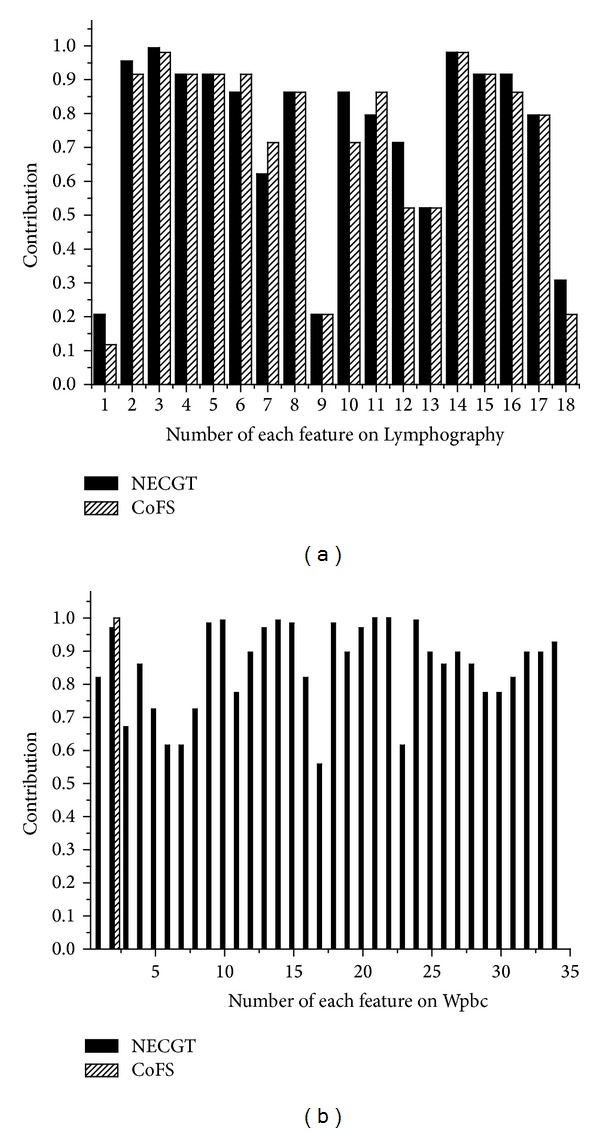
The contribution of each feature on Lymphography and Wpbc.

**Figure 4:**
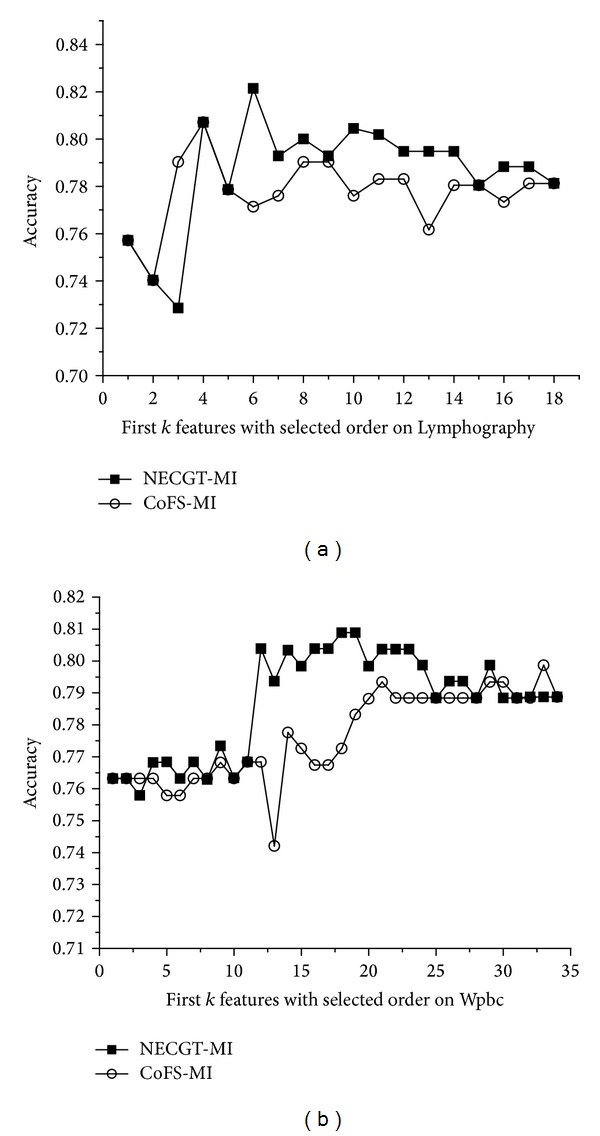
The results of NECGT-MI versus CoFS-MI. (a) The classification algorithm is CART; (b) the classification algorithm is RSVM.

Figure 3 shows the feature contribution which is evaluated by NECGT and CoFS.  Table 8 shows the features' order that the attributes are kept being added to the feature space. We can see the performance of first *k* features in Figure 4.

Some features on Lymphography get slightly different contribution scores because of the discretization. It makes the diversity of feature order.  Figure 4(a) shows that NECGT performs much better than CoFS except the third time iteration. NECGT-MI achieves the highest classification accuracy 82.14% where the selected feature subset is 13, 14, 2, 15, 3, and 16. The key reason is some information in Lymphography is lost by CoFS. Hu et al. [1] pointed out that there are at least two categories of structures lost in discretization: neighborhood structure and order structure in real spaces. For example, we know the distances between samples and we can get how the samples are close to each other in real spaces. In other words, it is unreasonable to measure similarity or dissimilarity with Euclidean distance as to categorical attributes in numerical methods. Therefore, it can be concluded that the discretization can lead to computational deviation of contribution evaluation, even if there is very little loss of information.

Then, we reflect on Wpbc. The contribution of most features is rated zero except feature 2 by CoFS. It can be concluded that the discretization makes serious distortion on Wpbc. As a consequence of this, CoFS-MI has to select the feature sequence in ascending order according to the feature number besides feature 2. Obviously, CoFS is inapplicable when the dataset is sensitive to discretization. In contrast, we can see that NECGT appears completely normal in the experiment. It shows that the nondiscretization method NECGT is suitable for more datasets.

For further comparison between NECGT and CoFS, we test the classification performance and running time on different datasets besides Lymphography and Wpbc.

As Experiment 1, the selected feature subset and the classification accuracy are displayed in Tables 9 and 10. NECGT-MI yields better performance than CoFS-MI in most cases. It can be concluded that NECGT is more efficient by avoiding discretization.  Table 11 shows the running time for each feature selection model. For example, computational overhead of NECGT and MI are 26.1 and 9.9, respectively, on Crx. Meanwhile, the running time for CoFS is 26.7. Compared with CoFS, NECGT is less time consuming. The main reason is that the process of discretization can take anywhere a fraction of a second to complete. On the other hand, we can see that the computational time of feature contribution has accounted for a considerable proportion in NECGT-MI (or CoFS-MI) model.

### 5.3. Discussion about Some Open-Ended Questions

The two experiments show the validity of the method in our study. NECGT indeed enhances the ability of classification of the attributes subsets. Nonetheless, there will be some open-ended questions if NECGT is applied to a real environment.

1. It is noticeable that the proposed method performs different performances for different classifiers on the same dataset. Consequently, for different application fields, a suitable classifier is also necessary. And this issue is one of the most important challenges in the application of artificial intelligence.
2. Although NECGT takes quite some time consequentially, the traditional methods are improved by NECGT. It necessary to consider which one is more important between high classification accuracy and fast computing in the practical application.
3. The validity of our model has been verified preliminarily on the UCI datasets. However, the large-scale dataset exists in the real environment. Consequently, the version of NECGT-SIGFD under distributed framework [18] is definitely worth exploring in the future work.

## 6. Conclusion and Future Work

Feature selection is an important preprocessing step in pattern recognition and machine learning. Traditional information-theoretic based selectors tend to ignore some features which as a group have strong discriminatory power but are weak as individuals. To overcome this disadvantage, we introduce a neighborhood entropy-based cooperative game theory framework to evaluate the contribution of each feature. The contribution of features is considered as another important factor for calculating the significance of features. Experimental results on UCI datasets show that the proposed method works well and outperforms traditional feature selectors at most cases. On the other hand, although CoFS also can be used for estimating the contribution of features by using Shannon's entropy, the major defect of CoFS is Shannon's entropy is only suitable for dealing with nominal data. Consequently, we redefine the redundancy, interdependence, and independence of features by using neighborhood entropy to avoid the loss of information caused by Shannon's entropy. Experimental results show that NECGT performs better than CoFS in most cases.

The future work could move along three directions. First, many other entropy models also can be used to calculate the relevancy of features, such as kernel entropy and fuzzy entropy. How to evaluate the interaction of these entropy modes is an important issue. Second, we will continue to construct the game theoretic-based feature selection model by adopting approximate Shapley value estimate technique [12]. Thirdly, the application of our model in the real environment is necessary. The version of NECGT-SIGFD under the distributed framework requires further attention.

## Figures and Tables

**Algorithm 1:**
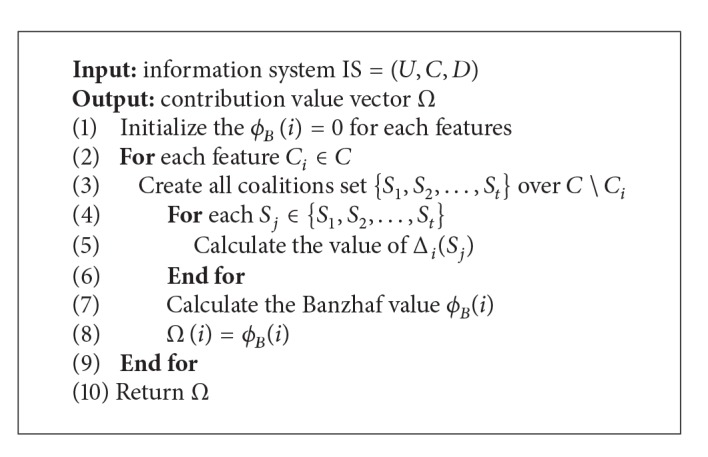
Feature contribution evaluation based on the Banzhaf value.

**Algorithm 2:**
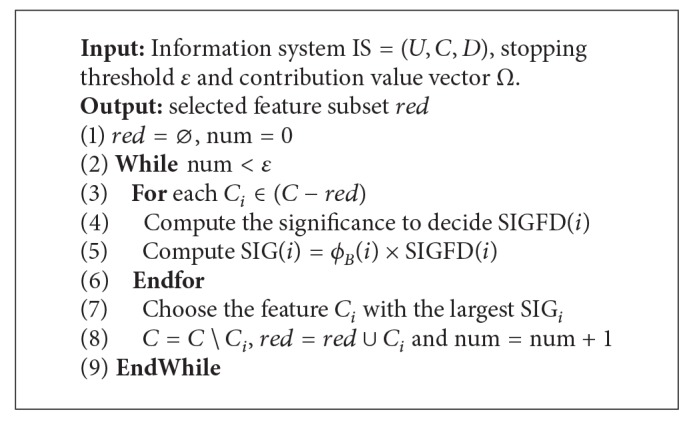
Feature selection with NECGT.

**Table 1:** Data description.

ID	Data	Samples	Features	Classes
1	Glass	214	9	7
2	Cardiotocography	2126	22	3
3	Wpbc	198	33	2
4	Crx	690	15	2
5	Hepatitis	155	19	2
6	Wine	178	13	3
7	Spectf	267	44	2
8	Lymphography	148	18	4
9	German	1000	20	2

**Table 2:** Order of feature selection on Glass.

Method	Order
mRMR	4, 7, 2, 3, 8, 1, 6, 5, 9
NECGT-mRMR	4, 7, 3, 1, 6, 2, 8, 5, 9

**Table 3:** Order of feature selection on different datasets.

Data	NECGT-mRMR	mRMR	NECGT-RS	RS
Lymphography	13, 5, 17, 8, 2, 15	13, 5, 18, 1, 9, 2	13, 2, 15, 14, 3, 16	13, 2, 15, 14, 10, 1

Crx	9, 14, 6, 8, 15	9, 15, 6, 11, 8	9, 6, 12, 1, 14	9, 10, 14, 6, 2

Cardiotocogrphy	6, 22, 11, 2, 1, 4, 5, 7, 19	6, 22, 11, 2, 18, 5, 7, 4, 8	5, 15, 4, 3, 16, 17, 11, 22, 1	6, 8, 13, 7, 1, 2, 3, 4, 5

Spectf	26, 34, 36, 24, 43, 28, 44, 8, 30, 10, 16, 15, 25, 14	26, 34, 10, 4, 40, 14, 7, 28, 6, 43, 30, 15, 32, 42	33, 37, 21, 38, 3, 5, 24, 27, 34, 13, 29, 20, 23, 36	26, 43, 3, 1, 2, 4, 5, 6, 7, 8, 9, 10, 11, 12

Hepatitis	12, 18, 17, 6, 11, 5	14, 12, 19, 17, 11, 6	15, 16, 8, 9, 3, 18	15, 16, 1, 2, 3, 4

**Table 4:** Classification accuracies based on CART (%).

Data	Raw data	NECGT-mRMR	mRMR	NECGT-RS	RS
Lymphography	78.31	82.86	70.00	82.14	79.03
Crx	84.92	84.50	84.93	81.88	81.17
Cardiotocography	85.94	85.74	85.69	75.11	83.22
Spectf	81.29	85.37	83.50	70.39	70.09
Hepatitis	82.33	84.17	77.33	78.50	72.33

Average	82.55	84.52	80.20	77.60	77.16

**Table 5:** Classification accuracies based on LSVM (%).

Data	Raw data	NECGT-mRMR	mRMR	NECGT-RS	RS
Lymphography	79.74	76.88	74.74	76.43	76.88
Crx	85.51	85.51	85.51	85.51	85.51
Cardiotocography	84.60	85.80	84.87	82.60	80.56
Spectf	84.65	84.63	84.23	79.41	79.41
Hepatitis	82.33	84.33	81.00	79.50	79.50

Average	83.36	83.43	82.07	80.69	80.37

**Table 6:** Classification accuracies based on RSVM (%).

Data	Raw data	NECGT-mRMR	mRMR	NECGT-RS	RS
Lymphography	55.52	84.03	72.14	76.17	75.71
Crx	69.14	84.50	84.21	83.34	86.37
Cardiotocography	79.54	85.23	85.14	83.12	82.56
Spectf	83.52	85.71	85.74	79.41	79.41
Hepatitis	87.00	82.50	81.83	78.83	77.50

Average	74.94	84.39	81.81	80.17	80.31

**Table 7:** A comparison of results.

Model	Win	Tie
NECGT-mRMR versus mRMR	12 : 2	1
NECGT-RS versus RS	8 : 3	4
NECGT-SIGFD versus SIGFD	20 : 5	5

**Table 8:** Order of feature selection.

Data	Model	Oder
Lymphography	NECGT-MI	13, 14, 2, 15, 3, 16, 5, 4, 8, 6, 10, 11, 17, 12, 7, 18, 1, 9
	CoFS-MI	13, 14, 15, 2, 3, 5, 6, 4, 8, 11, 16, 17, 7, 10, 12, 9, 18, 1

Wpbc	NECGT-MI	2, 14, 10, 34, 22, 24, 21, 15, 27, 9, 18, 20, 13, 12, 25, 33, 32, 19, 30, 4, 26, 28, 31, 16, 1, 11, 29, 5, 8, 3, 6, 7, 23, 17
	CoFS-MI	2, 1, 3, 4, 5, 6, 7, 8, 9, 10, 11, 12, 13, 14, 15, 16, 17, 18, 19, 20, 21, 22, 23, 24, 25, 26, 27, 28, 29, 30, 31, 32, 33, 34

**Table 9:** Order of feature selection on different datasets.

Data	NECGT-MI	CoFS-MI
Crx	9, 6, 12, 1, 14	9, 7, 6, 1, 12
German	1, 4, 7, 3, 11	1, 3, 4, 7, 6
Glass	3, 1, 5, 6	4, 1, 2, 7
Wine	9, 5, 3, 4, 8, 10	9, 5, 3, 4, 8, 1

**Table 10:** Classification accuracies (%) on the selected feature space.

Data	CART		LSVM		RSVM	
	NECGT-MI	CoFS-MI	NECGT-MI	CoFS-MI	NECGT-MI	CoFS-MI
Crx	81.88	81.47	85.51	85.51	83.34	80.57
German	72.40	71.82	70.00	70.00	70.30	70.90
Glass	71.68	71.18	52.52	50.39	53.77	58.45
Wine	86.67	86.60	87.78	83.89	91.11	86.67

Average	78.15	77.76	73.95	72.44	74.63	74.14

**Table 11:** Running time (seconds) for each feature selection model.

Data	MI	NECGT-MI	CoFS-MI
Crx	9.9	26.1 + 9.9	26.7 + 9.9
German	47.4	102.9 + 47.4	104.4 + 47.4
Glass	0.2	1.22 + 0.2	1.44 + 0.2
Wine	0.3	2.94 + 0.3	3.03 + 0.3
